# Assessment of Shoot Priming Efficiency to Counteract Complex Metal Stress in Halotolerant *Lobularia maritima*

**DOI:** 10.3390/plants12071440

**Published:** 2023-03-24

**Authors:** Alina Wiszniewska, Wojciech Makowski

**Affiliations:** Department of Botany, Physiology and Plant Protection, Faculty of Biotechnology and Horticulture, University of Agriculture in Kraków, Al. 29 Listopada 54, 31-425 Cracow, Poland

**Keywords:** ectoine, halophyte, hydrogen sulfide, multi-metal stress, nitric oxide, priming

## Abstract

The study investigated whether short-term priming supports plant defense against complex metal stress and multiple stress (metals and salinity) in halophyte *Lobularia maritima* (L.) Desv. Plants were pre-treated with ectoine (Ect), nitric oxide donor—sodium nitroprusside (SNP), or hydrogen sulfide donor—GYY4137 for 7 days, and were transferred onto medium containing a mixture of metal ions: Zn, Pb, and Cd. To test the effect of priming agents in multiple stress conditions, shoots were also subjected to low salinity (20 mM NaCl), applied alone, or combined with metals. Hydropriming was a control priming treatment. Stress impact was evaluated on a basis of growth parameters, whereas defense responses were on a basis of the detoxification activity of glutathione S-transferase (GST), radical scavenging activity, and accumulation of thiols and phenolic compounds. Exposure to metals reduced shoot biomass and height but had no impact on the formation of new shoots. Priming with nitric oxide annihilated the toxic effects of metals. It was related to a sharp increase in GST activity, glutathione accumulation, and boosted radical scavenging activity. In NO-treated shoots level of total phenolic compounds (TPC) and flavonoids remained unaffected, in contrast to other metal-treated shoots. Under combined metal stress and salinity, NO and H_2_S were capable of restoring or improving growth parameters, as they stimulated radical scavenging activity. Ect and H_2_S did not exert any effect on metal-treated shoots in comparison to hydropriming. The results revealed the stimulatory role of nitric oxide and low doses of NaCl in combating the toxic effects of complex metal stress in *L. maritima*. Both NO and NaCl interfered with thiol metabolism and antioxidant activity, whereas NaCl also contributed to the accumulation of phenolic compounds.

## 1. Introduction

Environmental pollution with toxic metals is a serious threat to the functioning of ecosystems and human health. One of the main sources of metal contamination in the soils is metalliferous mining which elevates the concentration of toxic compounds above the level acceptable for sustaining undisturbed growth and development of soil organisms, including plants that inhabit polluted sites [1,2]. The most hazardous are ballast elements, such as Cd, Pb, Hg that usually do not have any crucial biological function but exert a wide spectrum of negative effects on cellular and whole-plant levels. Also, although numerous metal ions, including Cu, Zn, Mn, Co, and Ni, are essential to plants, in excessive amounts these elements become as detrimental as ballast metals [3]. They inhibit plant growth by damage to cellular components and disorganization of their functioning, and force plants to activate defense mechanisms to counteract oxidative stress. Often several different metals are simultaneously present in post-mining contaminated soil, and their toxic action may cumulate. Plants are then subjected to so-called multi-metal stress [4,5]. Apart from the abundance of toxic metals, post-industrial soils are often excessively salinized [6]. It contributes to the development of multi-stress conditions, further altering the physiological response of the inhabiting plant species, including those tolerant to metal toxicity that could be used for soil cleanup via phytoremediation [7,8]. Conditions of both multi-metal and combined metal and salinity stress can be easily applied in in vitro culture to reproduce environmentally-encountered conditions in post-mining areas [9,10,11,12,13].

Phytoremediation and technologies for post-industrial landscape revegetation require the selection of plant species that are capable of accumulating soil contaminants in their organs and are tolerant to their toxicity [14]. The potential of using natural extremophytic flora inhabiting metalliferous soils is often limited due to the considerable susceptibility of such ecotypes to salinity [11,15]. Instead, an exploitation of salt-tolerant species is proposed for cultivation in those post-industrial areas, where soils are contaminated with toxic metals [16,17]. Halophytes are equipped with numerous mechanisms that enable plant survival in extreme conditions, particularly high salinity, xerothermic environment, or low temperatures [18,19]. They can maintain ionic homeostasis in the cells, regulate osmotic conditions, exclude toxic compounds via specialized trichomes, as well as prevent oxidative damage by an efficient antioxidant system. These adaptations are beneficial also to counteract the toxic effects of trace metal uptake and accumulation [20,21,22]. Sweet alyssum *Lobularia maritima* (L.) Desv. is a perennial herb native to the Mediterranean basin inhabiting coastal zones, dunes, and scrublands [23]. Apart from its ornamental character, the species is a facultative halophyte that exhibits a wide range of stress tolerance, and grows on saline, dry, poor, or contaminated land [24]. *L. maritima* has a short growth cycle and produces high biomass, which is advantageous for phytoremediation purposes. One of the characterized ecotypes was collected from an iron-cobalt tailings site in the Panzhihua region, China, and was further used for Co phytoextraction [25]. This halotolerant species is taxonomically related to salt-sensitive *Arabidopsis thaliana* [26], which facilitates molecular and genetic engineering studies. Genes encoding several stress-associated proteins in *L. maritima* served as transgenes for stress tolerance improvement in non-tolerant species. The promoter of LmSAP zinc finger was used to enhance tolerance to Al, Cu, Cd, Zn, salinity, and cold in rice [27], while the thioredoxin-h2 gene encoding plasma membrane protein Trx facilitated tobacco survival under salinity, osmotic and oxidative stress [28]. There is also an interest in the exploitation of *L. maritima* as a source of bioactive compounds, food additives, and pharmaceuticals [29]. Recently, bioactive polysaccharides extracted from this species were applied as biostimulators for wheat grown in saline soil and provided tolerance to NaCl by amelioration of oxidative damage [30]. However, nothing is known about the responses of this promising species to complex multi-metal stress as well as about the impact of salinity on metal toxicity. The possibilities of boosting species resilience to multi-metal stress using chemical priming have not been evaluated to date as well. This approach was found efficient in the stimulation *L. maritima* germination under severe salinity conditions [31].

Plant priming relies on short pretreatment with various chemical, physical, or biological stimuli, to reach higher survivability and ameliorate defense responses in subsequent stress conditions. In contrast to acclimation, which develops during prolonged chronic exposure to stress, and requires stable metabolic adjustment, priming is reversible and allows for resource-saving [32]. In the case of metal stress, short-term preconditioning induces a temporary primed state that is reflected in enhanced abilities to counteract metal toxicity. Stress resilience acquired during priming depends on the nature of the priming agent and encountered challenge [33]. Small signaling compounds that trigger tolerance to metal toxicity, such as reactive oxygen, nitrogen, and sulfur species, intensify direct metal detoxification and prevention of oxidative impairments [34]. Exogenously applied gasotransmitters: NO and H_2_S, influence the pool of their endogenous forms, boost antioxidant activity, and affect the accumulation and distribution of toxic ions in plant organs [32,35]. Due to their gaseous nature, priming with NO and H_2_S is conducted with the use of donor compounds. Seeds, seedlings, and explants are soaked in water solutions containing donors, which are taken up and metabolized to release active compounds. The most frequently used donors for NO and H_2_S are sodium nitroprusside (SNP) and sodium hydrosulfide (NaHS), respectively [32]. GYY4137 (morpholin-4-ium 4-methoxyphenyl(morpholino) phosphinodithioate) is a novel, stable donor that slowly releases H_2_S directly to the plant organs, without excessive H_2_S emission to the atmosphere [36]. Although it is widely used in agriculture, to date GYY4137 has not been tested as a priming agent toward metal stress tolerance. Compatible solutes, such as proline and betaines, are also suitable priming agents [37,38]. A prospective cyclic amino acid derivative is ectoine ((S)-2-methyl-1,4,5,6-tetrahydropyrimidine-4-carboxylic acid), a natural compound excreted by numerous halophilic and halotolerant bacteria. It is a compatible solute, osmoprotectant, enzyme, and membrane stabilizer, acting as a chemical chaperone for structural proteins and as a DNA protectant [39,40,41]. Recently ectoine was reported to regulate water status in Cd-stressed maize, facilitating detoxification [42]. However, nothing is known about its impact on cultured plants subjected to multi-metal treatment.

This study aimed at evaluating the effect of shoot priming with signaling and stress-protecting compounds on the growth and detoxification response of halotolerant *Lobularia maritima* in various suboptimal environments: under complex metal stress (simultaneous exposure to 700 µM ZnSO_4_, 16 µM CdCl_2_, and 3 µM PbCl_2_), low salinity (20 mM NaCl), as well as under combined action of multi-metal stress and low salinity.

We hypothesized that applied priming compounds may influence plant immunity through the enhancement of synthesis and activity of metabolites involved in plant defense response. Another hypothesis assumes some of them can deliver precursors for the synthesis of defense compounds and act as signaling molecules simulating the action of the mild stressor. The main interest was put on the detoxification and antioxidant properties of metabolites involved in stress response, mainly the accumulation of thiol compounds and phenolic compounds. Thiol-containing species are important indicators of plant defense against toxic metals, since they take part in the formation of chelate complexes with metals, reducing their toxicity in the cytosol, or sequestering them in vacuoles [43]. However, in metal-tolerant species, this mode of detoxification is often avoided, probably due to high metabolic costs [10,44]. The importance of thiol compounds in reaction to metal stress in halophytes is still unclear, although fluctuations in glutathione level and phytochelatin synthesis were observed in some species [45,46]. Phenolic compounds constitute the largest group of plant secondary metabolites allowing for the acclimation of plants to unfavorable environmental conditions [47]. Depending on the chemical structure of phenolic compounds, they have the ability to electron-donation and radical scavenging, which inhibits the action of reactive oxygen species (ROS) synthesized under metal stress [12]. Many plant species characterized by a naturally high concentration of phenolic metabolites or unique phenolic composition (ability to the synthesis of some rare phenolic derivatives, e.g., 1,4-naphtoquinones) are capable of sustaining growth on metal-contaminated soil, such as *Pontechium maculatum* [48]. In this study we also analyzed the activity of glutathione S-transferase, a key enzyme combining the ability to detoxification and radical scavenging [49,50], to facilitate an understanding of observed plant responses.

## 2. Results and Discussion

### 2.1. Priming Effect on Growth Response of L. maritima Varies in Different Stress Conditions

**Figure 1:**
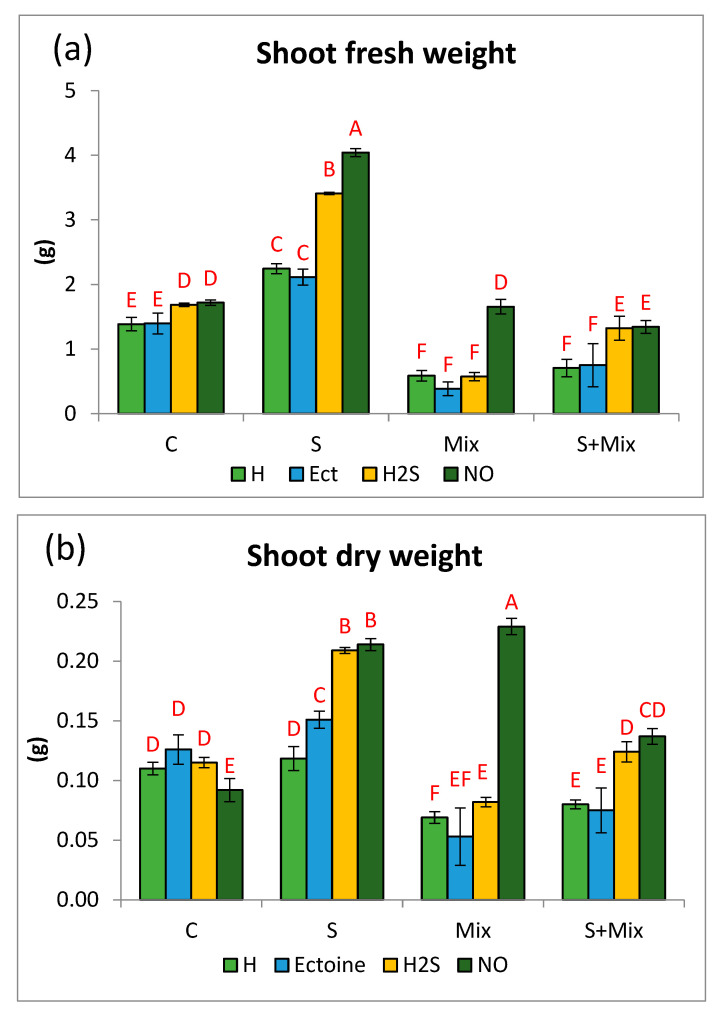

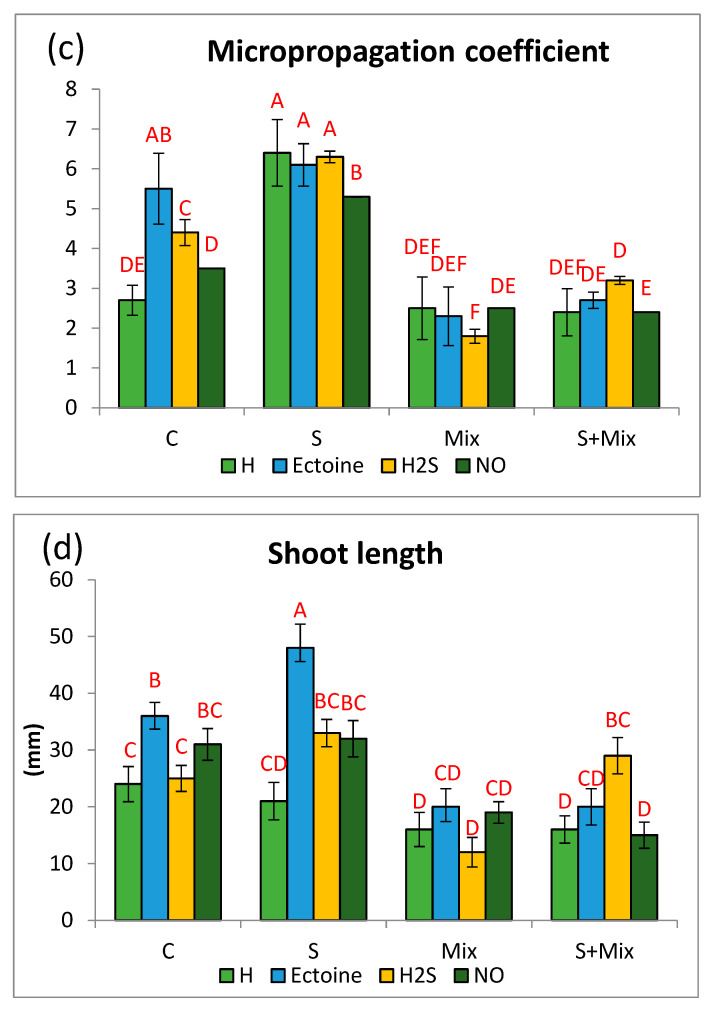
Growth parameters of *Lobularia maritima* shoots subjected to priming prior to stress exposure: (**a**) shoot fresh weight; (**b**) shoot dry weight; (**c**) micropropagation coefficient; (**d**) shoot length. Upper case letters indicate statistical significance of means acc. two-way ANOVA. Applied primers: H—water (hydropriming), Ect—ectoine, H2S—hydrogen sulfide donor GYY4137, NO—nitric oxide donor sodium nitroprusside. Treatments: C—control; S—low salinity (20 mM NaCl); Mix—metals: 0.7 mM ZnSO_4_, 16 µM CdCl_2_, 3 µM PbCl_2_; S + Mix—salinity combined with metals.

This study showed that the effects of priming on plant growth responses are strongly dependent on experimental conditions, namely the presence or absence of stress factors. In non-stressful conditions (control conditions, C) priming with H_2_S and NO slightly, but significantly, increased the fresh biomass of cultured *L. maritima* shoots (Figure 1a). Ectoine (Ect) had no effect on this parameter in comparison to control hydropriming. At low salinity (S) FW increased 1.5 times after priming with H_2_S and doubled after NO treatment. FW increased also in shoots primed with water and Ect in comparison with non-stressful conditions (Figure 1a). Exposure to metals (Mix) reduced the fresh weight of shoots treated with water, Ect, and H_2_S (by 61, 73, and 66%, respectively). Only NO was capable of restoring FW to the level noted in a non-stressful environment (Figure 1a). When metals were combined with low salinity (S + Mix), NO and H_2_S priming boosted FW accumulation, whereas hydropriming and Ect were ineffective. FW was there as low as under metal stress (Figure 1a).

In non-stressful conditions priming either had no effect (Ect and H_2_S) or reduced dry biomass accretion (NO) (Figure 1b). In turn, at low salinity (S) priming caused a significant increase in shoot DW. In shoots treated with H_2_S and NO obtained DW was twice as high as in the control. In Mix treatment, DW dropped in shoots primed with water, Ect, and H_2_S in comparison with respective priming agents in non-stressful conditions. Under metal stress priming with NO substantially induced DW (255% of the value for NO priming in non-stressful conditions), and these shoots had the highest DW from among all experimental treatments (Figure 1b). Under combined stresses (S + Mix) NO and H_2_S were capable of increasing DW accretion in the shoots to a level comparable to control shoots in non-stressful conditions (Figure 1b). 

In non-stressful conditions priming with Ect and H_2_S increased the formation of new shoots, expressed as micropropagation coefficient (MC). MC amounted to 5.5 and 4.4 for Ect and H_2_S, respectively, in comparison with 2.7 in hydroprimed shoots (Figure 1c). Low salinity (S) itself was stimulatory for shoot proliferation. MC increased to 6.4 in the case of control hydropriming. Ectoine and H_2_S priming also increased MC to 6.1 and 6.3, respectively (Figure 1c). 

In metal-containing treatments (Mix and S + Mix) micropropagation coefficients were comparable among the applied priming agents. Metals, either alone or combined with low salinity, did not deteriorate MC in relation to control in non-stressful conditions (Figure 1c). In the majority of priming variants, metals combined with salinity acted similarly to metals applied alone. No significant improvement in shoot proliferation was achieved by any priming treatment (Figure 1c). 

Shoot length in non-stressful conditions was elevated by Ect, while H_2_S and NO had no impact on this parameter (Figure 1d). In the presence of metals (Mix and S + Mix), shoot length was reduced after hydropriming. When metals were applied alone (Mix), Ect and NO priming were capable of restoring shoot elongation. When metals were combined with salinity (S + Mix), the ameliorating effect was exerted by Ect and H_2_S. Shoot length was the highest after Ect priming (36 and 48 mm in non-stressful and low salinity conditions, respectively) (Figure 1d). 

Although priming is usually applied to improve plant growth in the unfavorable environment [51], signaling molecules of priming character are also used for yield intensification under optimal conditions. Application of H_2_S-releasing GYY4137 was reported to increase the root harvest weight of radish, and the height and weight of pea plants [52]. NO-donor, SNP, enhanced in vitro shoot proliferation of wild *Malus* species [53], and medicinal plants, such as *Sideritis reaseri* [54] and *Canscora diffusa* [55]. In *Zea mays,* ectoine ameliorated relative growth rate not only under Cd-stress, but also in non-stressful conditions [42]. Here in *Lobularia maritima* explants were subjected to priming agents only for short time, but their beneficial action was maintained for several weeks of culture duration, resulting in efficient multiplication (for Ect) and biomass accretion (for H_2_S, and NO). 

Metal stress deteriorated *L. maritima* growth, however, proliferation capacity was unaffected. Growth inhibition was rather expected, since the metal mixture added to the medium contained two toxic ballast elements Pb and Cd, and a suboptimal amount of Zn. In a similarly modeled environment Muszyńska et al. (2018) [10] observed reduced biomass accretion in both metallotolerant and non-tolerant ecotypes of *Alyssum montanum*. Metallicolous ecotype maintained the same proliferation ability under metal exposure as in a non-contaminated medium. A similar response was achieved here in the culture of *L. maritima*, suggesting that this species has some potential to counteract the toxicity of ballast metals. Among priming agents, only NO stimulated biomass accretion in *L*. *maritima* under metal stress. Exogenous nitric oxide was previously shown to ameliorate growth parameters in plants subjected to Cd [56,57], however, the beneficial effects mostly involved the restoration of growth to the level obtained in Cd-untreated control plants. The substantial increase of biomass accumulation after NO priming in *L. maritima* may indicate on activation of some NO-dependent defense pathway that could involve cell wall rearrangements for efficient binding of toxic ions [57], which is then reflected in higher plant biomass. 

Salinity may exert negative effects on halotolerant species at the early stages of their life cycle [31], as well as affect their in vitro regeneration potential [58]. In this study low salinity had a stimulatory effect on *L. maritima* growth, and it was rather expected as this species is a facultative halophyte [24]. Therefore, a saline environment should not be considered stressful for *L. maritima*. Growth intensification observed in *L. maritima* could be attributed to the halophilic feature that consists of NaCl-induced capacity for cell division and accumulation of required energy supply [59]. 

Salinity applied simultaneously with metals ameliorated the growth response of *L*. *maritima* in comparison with single metal stress. The beneficial effects of NaCl on cell differentiation and division were somehow suppressed by the presence of metallic ions, since shoot proliferation was not improved. In this environment, H_2_S and NO were efficient primers that further facilitated *L. maritima* growth. Exogenous H_2_S and NO are widely reported to counteract the toxic effects of trace metals [60,61,62,63]. Both gasotransmitters are expected to be involved in plant cross-adaptation and support survival under multiple stresses [64,65]. However, evidence explaining the mechanisms of their combined action under multiple stresses is yet sparse, and the results of our study may help to uncover some of such effects on a biochemical level.

### 2.2. Detoxification Machinery in L. maritima Is Promoted Mostly by NO Donor

#### 2.2.1. Glutathione S-Transferase Activity

**Figure 2:**
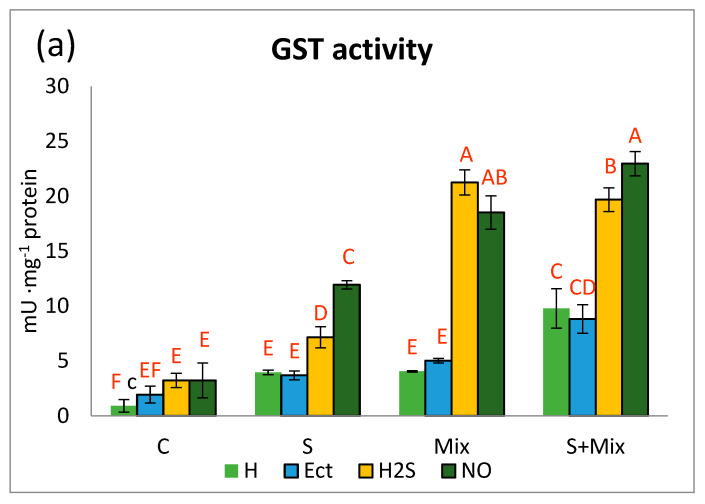

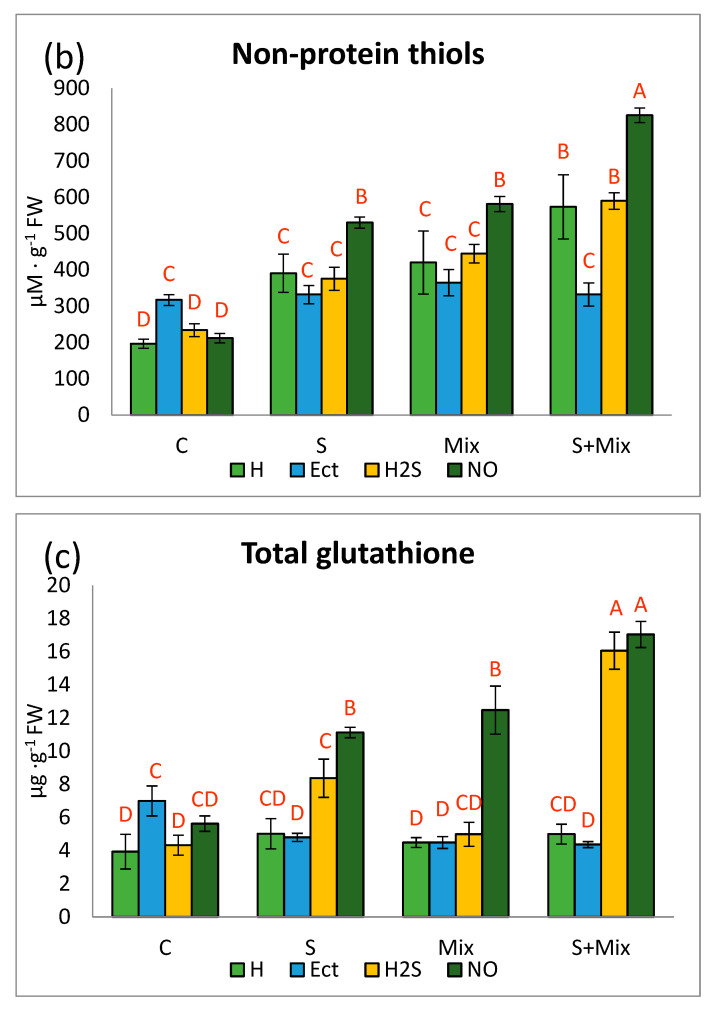
Components of the detoxification system in *Lobularia maritima* subjected to priming prior to stress exposure: (**a**) activity of glutathione S-transferase GST; (**b**) the content of non-protein thiols; (**c**) total content of glutathione GSH. Upper case letters indicate statistical significance of means acc. two-way ANOVA. Applied primers: H-water (hydropriming), Ect-ectoine, H2S-hydrogen sulfide donor GYY4137, NO-nitric oxide donor sodium nitroprusside. Treatments: C-control; S-low salinity (20 mM NaCl); Mix-metals: 0.7 mM ZnSO_4_, 16 µM CdCl_2_, 3 µM PbCl_2_; S + Mix-salinity combined with metals.

As an element of detoxification machinery in *L. maritima* we have evaluated the activity of the GST enzyme. This protein catalyzes the formation of glutathione conjugates with various ligands, including internal toxins, xenobiotics, and toxic metal ions, thus detoxifying the cellular environment [66]. In *L. maritima* GST activity was low in non-stressful conditions (C) and did not exceed 4 U·mg protein^−1^ in any treatment. H_2_S and NO slightly increased it in comparison with hydropriming and Ect. The activity always rose in stress conditions (Figure 2a). Comparable GST activity was determined at low salinity (S) and metals (Mix) after hydropriming and Ect. Particularly pronounced increments of GST activity were noted in shoots primed with H_2_S and NO. In the presence of metals (Mix), the activity rose from 4.06 U · mg protein^−1^ in hydropriming to 21.25 U · mg protein^−1^ in H_2_S-primed shoots, and to 18.51 U · mg protein^−1^ in NO-primed ones. When metals and salinity were combined (S + Mix), GST activity amounted to 19.61 and 22.96 U · mg protein^−1^ in the case of H_2_S and NO priming, respectively. Also priming with water and Ect enhanced GST activity in S + Mix treatment (Figure 2a). 

In the metal-tolerant ecotype of *Alyssum montanum* elevation of GST activity did not occur under metal treatment, suggesting it was on a stable constitutive level [10]. The increases in GST activity in *L. maritima* may be related to enhanced expression of the gene encoding GST protein, as in halophyte *Salicornia brachiata*, where the GST gene promoter is known to possess stress regulatory motifs [67]. In all experimental conditions evaluated in this study, GST activity rapidly increased after priming with H_2_S and NO. Protecting effect of H_2_S against Cr stress in maize was reported to rely on efficient detoxification machinery, including higher GST activity [61]. In contrast, the application of NO donor, SNP, had no effect on GST activity in As-treated wheat [68]. This could be attributed to the existence of several classes of distinct functionalities among GST enzymes. GST detoxification capability includes radical scavenging, and thus these enzymes contribute to general antioxidant response in stressed plants [49,50]. Therefore, boosted GST activity after H_2_S and NO priming could result from the intensification of antioxidant reactions aimed at balancing ROS overproduction under stress. More pronounced detoxification in *L. maritima* was induced by H_2_S and NO priming under metal exposure than under low salinity, corroborating the findings that both gasotransmitters are beneficial regulators of metabolic adjustment under metal stress [69,70].

#### 2.2.2. The Content of Non-Protein Thiols (NPT)

Synthesis and accumulation of sulfur-containing compounds is a defense strategy to counteract metal toxicity in the cytoplasm. Metal ions are bound to thiol groups, and as their intracellular pool decreases due to sequestration in the vacuole, cytoplasmic components are protected [71,72]. The total pool of thiol compounds in *L. maritima* varied depending on the culture environment and applied priming treatment. In non-stressful conditions, only Ect stimulated thiol accumulation (Figure 2b). As this pool remained stable under all stressful conditions, NPT elevation under Ect treatment was unlikely a stress response, but some uncovered beneficial effects of Ect. Similarly, considering the pool of glutathione, which is a major non-protein thiol, we observed that Ect induced its accumulation in non-stressful conditions, but has no effect when salinity or metal stress were applied. This is in line with a recent report on Cd-stressed maize, where Ect did not counteract a depletion of the glutathione pool [41]. Currently, there are scarce reports examining the role of Ect on thiol pool and metabolism, so further studies are required to reveal the mechanism of such a response. 

Both low salinity and metals had a comparable impact on the non-protein thiols pool (Figure 2b). When these stressors were applied separately, the highest increase in thiol content occurred after priming with NO. Similar improvement in NPT content was also reported in *Oryza sativa* stressed with As [73]. H_2_S and hydropriming also stimulated NPT accumulation. Significant elevation of thiol accumulation occurred when metals were combined with low salinity (Figure 2b). The highest content of thiols, exceeding 800 µM·g^−1^ FW (four times as much as in non-stressful conditions), was determined in shoots primed with NO (Figure 2b). This reflects an increased accumulation of the compounds that can be used for chelating and sequestering metal ions, most probably phytochelatins [74]. In *L. maritima* NPT level increased also under low salinity. It is interesting, since, as described above, we did not consider low salinity stressful for this species. Therefore, higher thiol accumulation may be related to efficient and balanced sulfur assimilation and metabolism in this halophyte [75]. 

#### 2.2.3. Glutathione Content

The total pool of glutathione was determined as a separate parameter, as this compound plays a pivotal role in stress responses, contributing to both detoxification and antioxidant capacity. In a non-stressful environment, an overall concentration of glutathione was low, and, as mentioned above, changed only after Ect priming (increase to 7.00 µg·g^−1^ FW) (Figure 2c). It can be postulated that in *L. maritima* glutathione is not a major thiol accumulated in the presence of low salinity or metals, and other thiols are massively synthesized instead. The stability of glutathione content, particularly under metal stress, is a rather uncommon reaction, but it was described also earlier in *Daphne jasminea* [76]. Usually GSH content depletes, as it is utilized for phytochelatin synthesis [77]. Metal detoxification in *L. maritima* is likely to be performed by other compounds, for instance by metal-binding proteins that are abundant in this species [27,28,78].

Considering the priming impact on the GSH pool, only NO caused its elevation under metal stress, whereas when low salinity was applied, also H_2_S exerted a stimulatory effect. The highest impact of both priming agents was manifested under combined stresses. In H_2_S-primed shoots, GSH content doubled at salinity alone, while increased four times when salinity was combined with metals (Figure 2c). Under metal stress the highest GSH accumulation occurred after NO priming (12.48 µg·g^−1^ FW), whereas under combined stresses both H_2_S and NO increased GSH levels (16.06 and 17.04 µg·g^−1^ FW, respectively) (Figure 2c). It was in line with an elevation of total NPT content. We suggest that NO and H_2_S induced overall synthesis of thiol compounds, with some portion of them designed for detoxification via ion sequestration, and the other pool addressed for the annihilation of oxidative impairments [70,79]. To elucidate the precise role of these gasotransmitters in thiol metabolism in stressed *L. maritima*, further studies could focus on distinguishing compounds constituting two functional pools, and involve the determination of protein-bound thiols, including the level of protein glutathionylation.

### 2.3. Contribution of Phenolic Compounds as Non-Enzymatic Antioxidants in Priming-Affected Stress Response

#### 2.3.1. Phenolic Profile

**Figure 3:**
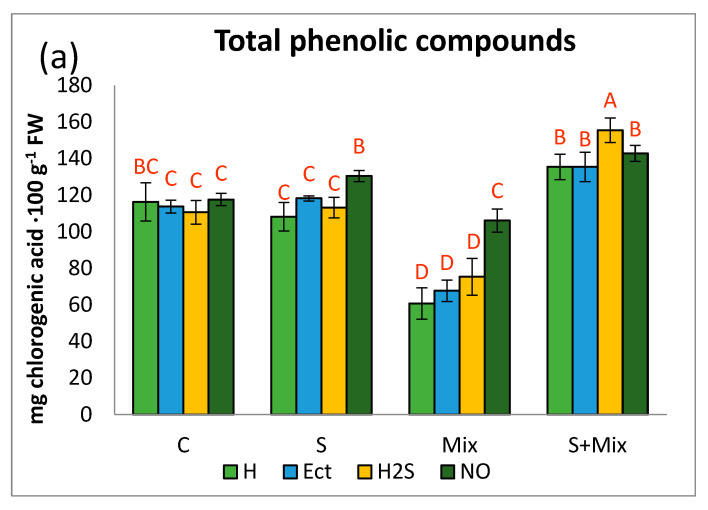

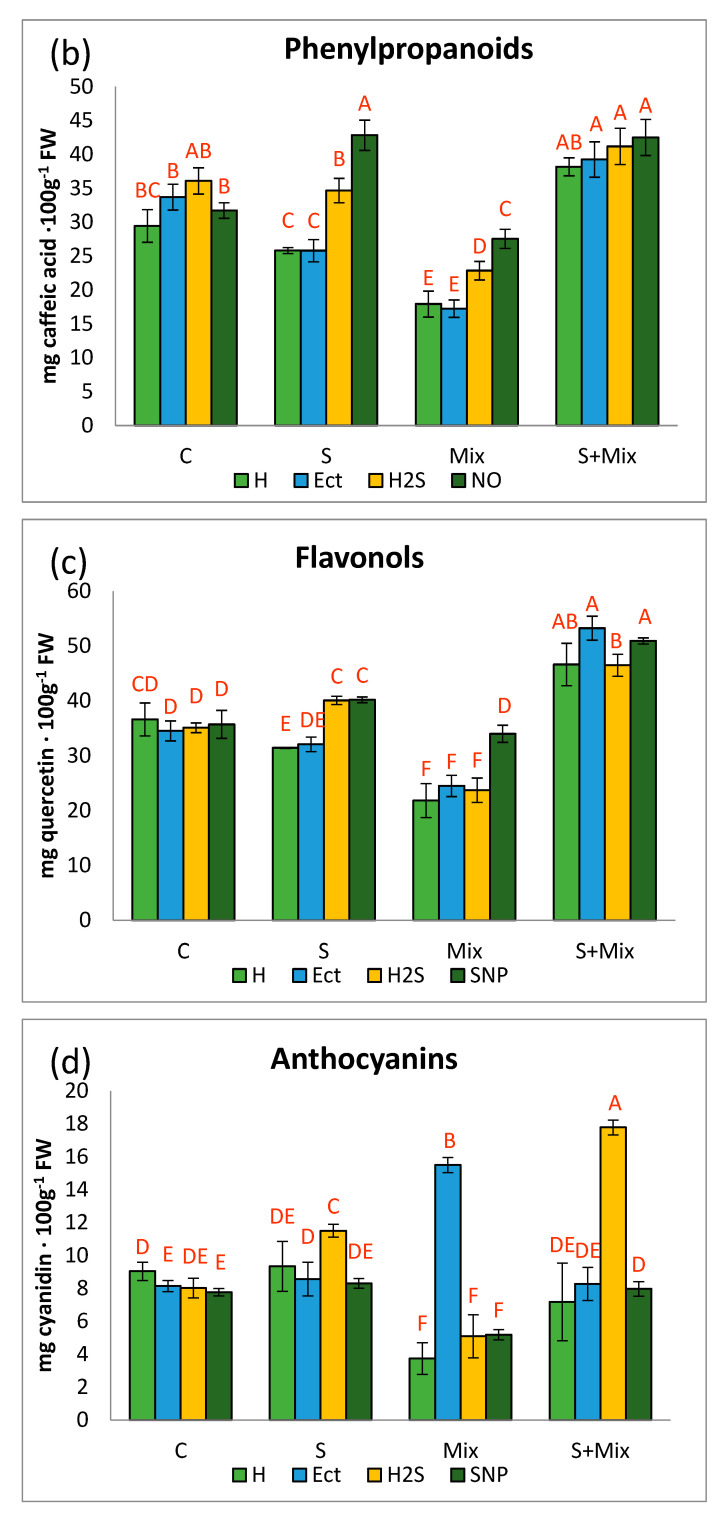
Phenolic profile in *Lobularia maritima* shoots subjected to priming prior to stress exposure: (**a**) total phenolic content; (**b**) phenylpropanoids; (**c**) flavonols; (**d**) anthocyanins. Upper case letters indicate statistical significance of means acc. two-way ANOVA. Applied primers: H—water (hydropriming), Ect—ectoine, H2S—hydrogen sulfide donor GYY4137, NO—nitric oxide donor sodium nitroprusside. Treatments: C—control; S—low salinity (20 mM NaCl); Mix—metals: 0.7 mM ZnSO_4_, 16 µM CdCl_2_, 3 µM PbCl_2_; S + Mix—salinity combined with metals.

Without stress, priming had no particular effect on the total content of phenolic compounds (TPC) in *L. maritima* (Figure 3a). Hydropriming did not affect this parameter also in treatments involving low salinity, alone or combined with metals. Priming with NO elevated TPC under low salinity in comparison with other priming agents in this environment. Application of metals decreased TPC substantially, apart from shoots subjected to NO priming, where TPC content was the same as in non-stressful conditions (Figure 3a). Under combined stresses, TPC was usually the highest, and H_2_S priming had the most significant impact on this pool (Figure 3a). Nitric oxide may increase plant tolerance to abiotic stresses through stimulation of the expression of genes involved in the phenylpropanoid pathway. Ge et al. (2019) [80] reported that priming with NO donor promoted the accumulation of precursors for phenolic compound synthesis. In *L. maritima* TPC was usually the highest under combined stresses, and H_2_S priming was the most impactful (Figure 3a). These results support observations of Montesinos-Pereira et al. (2016) [81], where hydrogen sulfide was recognized as the molecule promoting the synthesis of phenolic derivatives. Plants subjected to H_2_S priming may have more effective acclimation strategies to abiotic stress [82].

Phenylpropanoid and flavonol contents decreased in a presence of metals, while increased when metals were combined with low salinity (Figure 3b,c). The highest accumulation of these compounds occurred after priming with NO. More specifically, priming with NO and H_2_S boosted phenylopropanoid content under low salinity (S) and combined stresses (S + Mix) (Figure 3b). Flavonol content increased particularly under combined stresses (S + Mix), regardless of the priming agent (Figure 3c). It has been shown that under Pb exposure, the synthesis of phenylpropanoids or flavonoids, including flavonols, may decrease [83]. Plant priming may accelerate the synthesis of phenylpropanoids and flavonols, since it may alter pathways of secondary metabolism for ameliorating defense responses [84]. For example, NO-primed plants may have a higher level of phenolic derivatives, due to the enhanced activity of phenolic synthesis-related enzymes [80]. 

The pattern of anthocyanin content varied depending on the priming treatment and applied stress. In non-stressful conditions decline was noted after Ect and NO priming (Figure 3d). When low salinity was applied (S and S + Mix), H_2_S stimulated anthocyanin accumulation at most. Contrary to our results, Gohari et al. (2020) [85] showed that priming with NO donor-induced anthocyanins accumulation in *O. basilicum* L. When low salinity (S and S + Mix) was applied to *L. maritima*, H_2_S increased anthocyanin content at most. Under metal stress, only Ect induced anthocyanin accumulation, whereas other primers decreased it (Figure 3d). Anthocyanins are an important group of phenolic compounds involved in plant response in unfavorable conditions [86]. Sherin et al. (2022) [87] reported that various priming strategies can promote the synthesis of anthocyanins under stress. An interesting interconnection exists between anthocyanin sequestration in vacuoles and GST enzyme. It is postulated that GST acts as a carrier protein that non-enzymatically binds to flavonoids, without formation conjugates with glutathione [88]. The function of this protein is also to maintain flavonoid homeostasis, by recycling their oxidized forms, thus regenerating the antioxidant pool [50]. The precise role of priming agents on the relationships between elements of detoxification and antioxidant machinery under multi-stress is yet to be uncovered. 

#### 2.3.2. Radical Scavenging Activity

**Figure 4:**
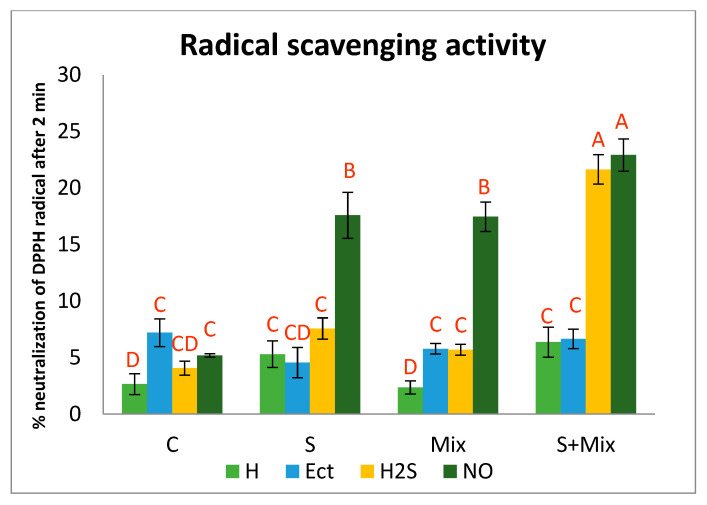
Radical scavenging activity of non-enzymatic antioxidants in *Lobularia maritima* shoots subjected to priming prior to stress exposure. Upper case letters indicate statistical significance of means acc. two-way ANOVA. Applied primers: H—water (hydropriming), Ect—ectoine, H2S—hydrogen sulfide donor GYY4137, NO—nitric oxide donor sodium nitroprusside. Treatments: C—control; S—low salinity (20 mM NaCl); Mix—metals: 0.7 mM ZnSO_4_, 16 µM CdCl_2_, 3 µM PbCl_2_; S + Mix—salinity combined with metals.

In control conditions (C) RSA of *L. maritima* hydroprimed shoots was considerably low (2.7%). Ect and NO increased this parameter slightly (Figure 4). When low salinity was applied (separately S and combined with metals S + Mix), RSA after hydropriming was at least twice as high as in non-stressful conditions (5.3 and 6.3% in S and S + Mix, respectively). The most prominent effect on RSA activity under stress had NO priming. RSA increased 3.5 times in NO-primed shoots, amounting to 17.6 and 17.5% in S and Mix, respectively. Further RSA increment, reaching 22.9%, occurred when metals were combined with salinity (Figure 4). In this environment, H_2_S was as effective as NO in the improvement of radical scavenging (Figure 4).

The spectrophotometric method with DPPH· radical enables us to estimate the ability of antioxidants present in plant tissues to reduce the radicals. In physiological research, this method can be used to determine the activity of plant-derived, non-enzymatic antioxidants [47]. It has been shown that Ect modulated the antioxidant system during the heat stress in planktonic crustacean *Daphnia magna* [89], whereas in maize exposed to Cd stress, it promoted the activity of enzymatic antioxidants, as well as influenced expression of the gene related to ascorbate and glutathione metabolism [41]. In our study, Ect increased RSA of hydroprimed shoots and shoots exposed to metal stress. It may relate to the fact that priming with Ect interfered with secondary pathways involved in the synthesis of antioxidant metabolites. RSA also increased in metal-treated *L. maritima* shoots primed with H_2_S, and in all those primed with NO. Montesinos-Pereira et al. (2016) [81] reported that hydrogen sulfide donor increases RSA and reduces power in *Brassica oleracea* L. ‘Bronco’. This priming agent also boosted the activity of the antioxidant system in *Cyclocarya paliurus*, facilitating acclimation to NaCl stress conditions [82]. Similarly, NO increased the antioxidant power of extract from *Triticum aestivum* L. cv. Chamran cultivated under salt stress [90]. Contrary to our findings, priming with NO did not affect antioxidant activity in non-stressed wheat plants. The status of antioxidants in plant tissue subjected to priming is dependent on NO concentration and priming time [90]. Treatment with NO can modify enzymatic and non-enzymatic antioxidant systems and facilitate defense response to metal stress in plants [91]. However, the effectiveness and specificity of NO priming are also dependent on plant species and type of stress [85,92], therefore precise mechanisms regulating this phenomenon in *L. maritima* should be distinguished.

## 3. Materials and Methods

### 3.1. Plant Material and Experimental Conditions

Shoots of *Lobularia maritima* (L.) Desv. were multiplicated in vitro on modified WPM medium [93] containing 12.3 µM N6-[2-isopentyl]adenine (2iP), 5.37 µM 1-naphthaleneacetic acid (NAA), 0.5 g·L^−1^ activated charcoal, and 20.0 g·L^−1^ sucrose. For the experiment, 10 mm long apical shoot fragments were explanted on a solid basal medium, and 5 mL of respective priming solution was poured onto the medium. Priming solutions included 0.1 mM ectoine ((S)-2-methyl-1,4,5,6-tetrahydropyrimidine-4-carboxylic acid) (treatments referred here as to Ect), 0.1 mM hydrogen sulfide donor-morpholin-4-ium4-methoxyphenyl phosphinodithioate (GYY4137, treatments referred here as to H_2_S), and 0.1 mM nitric oxide donor-sodium nitroprusside (SNP, treatments referred here as to NO). As priming control, distilled water was used (H, hydropriming). Priming solutions were freshly prepared, and filter sterilized prior to application. Shoots were primed for 7 days in a growth chamber and afterward were transferred onto test media supplemented with stress-inducing compounds: (1) for complex metal stress (Mix) medium was supplemented with a mixture of metals: 700 µM ZnSO_4_, 16 µM CdCl_2,_ and 3 µM PbCl_2_ according to Muszyńska et al. (2018) [10] (to reproduce conditions occurring on waste heaps formed after Zn-Pb ores mining and processing in Olkusz Ore Bearing Region, Poland); (2) for low salinity (S) medium was enriched with 20 mM NaCl, (3) for combined stresses (S + Mix) both 20 mM NaCl and the mixture of metals were added to the medium. Basal medium with no additives was used as a control in non-stressful conditions (C). Shoots were cultured for 4 weeks. 

For each experimental treatment, 5 Magenta boxes were used, with 6 explants in each box. The experiment was repeated thrice. 

### 3.2. Growth Parameters

After four weeks plant material was measured, weighted, and preserved in liquid nitrogen for subsequent biochemical analyses. Biometrical data included multiplication coefficient (calculated as the number of new shoots developed on initial explant), shoot length, and shoot fresh and dry weight. 

### 3.3. Glutathione-S-Transferase Activity

For GST activity 1-chloro-2,4-dinitrobenzene (CDNB) spectrophotometric assay was performed according to Habig and Jakoby (1981) [94]. Shoot samples (100 mg FW) were homogenized in 1 mL cold Tris-HCl buffer and centrifuged for 20 min at 15,000 rpm at 4 °C. Supernatant was used for the analysis. The reaction mixture consisted of 100 μL CDNB, 1.7 mL PBS reaction buffer, 100 μL 1 mM glutathione, and 100 μL extract. The reaction was conducted at 37 °C. Absorbances were read twice, without extract and after extract addition, at 340 nm at 0- and 2-min. GST activity was calculated on a basis of changes in absorbances and extinction coefficient for CDNB (9.6·10^−3^ mL/(nmol·cm)). Protein content was determined according to Bradford (1976) [95]. GST activity was then expressed as mU · mg protein^−1^ (activity of 1 mU is equal to 1 nmol of formed CDNB-glutathione conjugation product in 1 min at 1 mg protein).

### 3.4. Determination of Non-Protein Thiols

The content of non-protein thiols (total acid-soluble SH compounds) was determined spectrophotometrically according to De Vos et al. (1992) [96]. Samples (100 mg FW) were homogenized in 1 mL cold 5% sulfosalicylic acid with 6.3 mM diethy-lenetriaminepentaacetic acid (DTPA) (pH < 1) and centrifuged for 20 min at 15,000 rpm at 4 °C. Supernatant was used for the analysis. Reaction mixture (pH = 7) consisted of 630 µL 0.5 M K_2_PO_4_ reaction buffer, 300 µL extract, and 25 µL 10 mM 5,5′-dithiobis(2-nitrobenzoic acid) (DTNB). Absorbance was read after 2 min at 412 nm (30 °C). The content of thiols was calculated using extinction coefficient ε = 13,600.

### 3.5. Glutathione Determination

The total glutathione content in examined plants was measured using spectrophotometric method where 5,5-dithiobis(2-nitro-benzoic acid) (DTNB) acts as glutathione reductase (GR)-dependent reductor (Queval and Noctor 2007 [97], with modifications by Makowski et al. 2021 [98]). A total of 100 mg of fresh plant tissue was extracted in 1 mL 0.2 N HCl. The extract was centrifuged at 15,000 rpm for 15 min at 4 °C. A total of 0.5 mL of obtained supernatant was neutralized with 0.5 M NaOH in the presence of 50 μL 0.2 M NaH_2_PO_4_ (pH = 5.6) to reach a final pH between 5 and 6. Such pH is required for the optimal work of GR. The reaction mixture contained: 30 μL neutralized extract, 300 μL 0.2 M NaH_2_PO_4_ (pH = 7.5), 30 μL 10 mM EDTA, 30 μL 10 mM NADPH, 30 μL 12 mM DTNB, and 180 μL deionized water. The reaction was started by adding 30 μL GR (20 U·mL^−1^). The increase in absorbance value was monitored for 2 min at 412 nm. Calculations were made based on a standard curve prepared using glutathione (Sigma Aldrich, Germany). The results were expressed as µg of glutathione per g FW.

### 3.6. Radical Scavenging Activity

Stable free radical DPPH (2,2-diphenyl-1-picrylhydrazyl) was used to test the radical scavenging activity of *L. maritima* shoots (Pekkarinen et al., 1999) [99]. Shoot samples were extracted with 80% methanol. The changes in absorbance of DPPH· solution, following reduction of DPPH·, were measured at 517 nm at the moment of extract addition and after 10 min, using a Hitachi (Westford MA, USA) U-2900 spectrophotometer. The radical scavenging activity of extracts was expressed in % of reduced DPPH· radical by a unit of FW.

### 3.7. Phenolic Profile

Phenolic compounds (total phenolic content—TPC, phenylopropanoids, flavonols, and anthocyanins) in *L. maritima* shoots were determined using UV/VIS spectrophotometry (Fukumoto and Mazza 2000) [100]. Chlorogenic acid (CGA), caffeic acid (CA), and quercetin (QC) were used as standards for TPC, phenylopropanoids, and flavonols, respectively. Anthocyanin content was expressed as the cyanidin (CY), according to its molar extinction. Samples were ground with 1 mL of 80% methanol and centrifuged for 15 min at 3000× *g*. Supernatant was used for the analysis. The extract (0.25 mL) was mixed with 0.25 mL 0.1% HCl (in 96% ethanol) and 4.50 mL 2% HCl (in water) and after 30 min the absorbances at 280, 320, 360, and 520 nm were read (Hitachi U-2900 spectrophotometer). The content of phenolic compounds was expressed in mg of respective standard equivalents per 100 g FW.

### 3.8. Statistical Analyses

Data were statistically analyzed using STATISTICA 13.0 software (StatSoft, Tulsa, OK, USA). Two-way ANOVA and post hoc Tukey’s test were used to assess differences between *L. maritima* responses to priming treatments under optimal and stress conditions.

## 4. Conclusions

In this study, we describe for the first time how priming with ectoine, nitric oxide donor, and hydrogen sulfide donor may affect the growth of halophyte *Lobularia maritima* under multi-metal stress, applied alone, and combined with low salinity. Obtained results indicate that all tested primers may boost the defense response of examined species and help to deal with metal toxicity. Applied primers influenced the synthesis of protecting and detoxifying metabolites as well as enhanced glutathione-mediated detoxification. Primed shoots achieved higher tolerance for the harmful environment and the ability to mitigate the negative effects of stress. Our study showed that although priming facilitated response to multi-stress, its effectiveness depended on the type of primer and the type of stress. Further studies should focus on the detailed mechanism of primers’ action. The results also revealed that low salinity has a protective effect on *L. maritima* under multi-metal exposure, and this finding may facilitate the use of this species to revegetate soils in post-industrial areas.

## Data Availability

All data are included in the manuscript.
